# Supplementary material for: PvdL Orchestrates the Assembly of the Nonribosomal Peptide Synthetases Involved in Pyoverdine Biosynthesis in Pseudomonas aeruginosa
Source: Int J Mol Sci. 2024 May 30;25(11):6013. doi: 10.3390/ijms25116013 (PMC11172790; doi:10.3390/ijms25116013)
Supplement: Supplementary file 1 [file ijms-25-06013-s001.zip › ijms-2982383-supplementary.pdf]

Summary

The table S1 contains all the strains and plasmids used in this work. The Table S2 contains the oligonucleotides which were used in order to construct the plasmids. Figure S1 shows diffusion maps reconstructed for long and short jump distances.

Table S1

Strains and plasmids	Collection ID	Relevant characteristics	Source
Pseudomonas aeruginosa			
PAO1	PAO1	Wild-type strain	Stover et al. 2000
PvdA-eGFP	PAS180	eGfp chromosomally integrated	Gasser et al. 2015
eGFP-PvdL	PAS215	eGfp chromosomally integrated	Gasser et al. 2020
PvdI-eGFP	PAS216	eGfp chromosomally integrated	Gasser et al. 2020
PvdJ-eGFP	PAS471	eGfp chromosomally integrated	Gasser et al. 2020
eGFP-PvdD	PAS214	eGfp chromosomally integrated	Gasser et al. 2020
PvdA-PAmCherry	PAS405	PAmCherry chromosomally integrated	Gasser et al. 2015
PAmCherry-PvdL	PAS406	PAmCherry chromosomally integrated	This work
PvdI-PAmCherry	PAS407	PAmCherry chromosomally integrated	This work
PvdJ-PAmCherry	PAS560	PAmCherry chromosomally integrated	This work
PAmCherry-PvdD	PAS823	PAmCherry chromosomally integrated	This work
eGFP-PvdL / PvdJ-mCherry	PAS554	eGfp and mCherry chromosomally integrated	This work
PvdJ-eGFP / mCherry-PvdL	PAS555	eGfp and mCherry chromosomally integrated	This work
eGFP-PvdL / mCherry-PvdD	PAS556	eGfp and mCherry chromosomally integrated	This work
eGFP-PvdL / PvdI-mCherry	PAS557	eGfp and mCherry chromosomally integrated	This work
PvdJ-eGFP / mCherry-PvdD	PAS559	eGfp and mCherry chromosomally integrated	This work
Escherichia coli			
TOP10		F- mcrA Δ(mrr-hsdRMS-mcrBC) ϕ80lacZΔM15 ΔlacX74 nupG recA1 araD139 Δ(ara-leu)7697 galE15 galK16 rpsL(StrR) endA1 λ	Invitrogen
Plasmids			
pEXG2	pEXG2	Allelic exchange vector with pBR origin gentamicin resistance, sacB	Rietsch et al., 2005
pEXG2 PAmCherry-pvdL	pAF12	pEXG2 carrying the sequence to insert a PAmCherry tag in Nter of pvdL	This work
pEXG2 pvdI-PAmCherry	pAF11	pEXG2 carrying the sequence to insert a PAmCherry tag in Cter of pvdI	This work
pEXG2 pvdJ-PAmCherry	pTS01	pEXG2 carrying the sequence to insert a PAmCherry tag in Cter of pvdJ	This work
pEXG2 PAmCherry-pvdD	pTS04	pEXG2 carrying the sequence to insert a PAmCherry tag in Nter of pvdD	This work
pME3088 eGFP-PvdL	pVEGA16	pME3088 carrying the sequence to insert a eGFP tag in Nter of pvdL	Gasser et al. 2020
pME3088 pvdJ-mCherry	pLG45	pME3088 carrying the sequence to insert a mCherry tag in Cter of pvdJ	Gasser et al. 2020
pEXG2 pvdJ-eGFP	pVEGA30	pEXG2 carrying the sequence to insert a eGFP tag in Cter of pvdJ	Gasser et al. 2020
pME3088 mCherry-pvdL	pVEGA13	pME3088 carrying the sequence to insert a mCherry tag in Nter of pvdL	Gasser et al. 2020
pME3088 mCherry-pvdD	pLG47	pME3088 carrying the sequence to insert a mCherry tag in Nter of pvdD	Gasser et al. 2020
pME3088 pvdI-mCherry	pLG42	pME3088 carrying the sequence to insert a mCherry tag in Cter of pvdI	Gasser et al. 2020

Table S1: Strains and plasmids used in this work.

Table S2

Oligonucleotides	Sequence (5' to 3')	Used to construct plasmids
PvdL-HindIIIFN	AAAAAGCTTTTCGGCGAGGCCCTGCATACCG	pAF12
Overlap mCher/PvdL ATG-700 Rev	CCTCCTCGCCCTTGCTCACCATCATGTGTTTTCCTGCCTG	pAF12
PAmCherry forwad	GTGAGCAAGGGCGAGGAGG	pAF12 / pAF11 / pTS01 / pTS04
eGFP Rev	CTTGTACAGCTCGTCCATGC	pAF12 / pAF11 / pTS01 / pTS04
Overlap mCher/PvdL ATG+700 For	GCATGGACGAGCTGTACAAGGACGCCTTCGAACTTCCCACC	pAF12
PvdL-XhoIRN	TTTCTCGAGTACGCCGCTGAAGATCGGTTG	pAF12
PvdI-XhoIFC	AAACTCGAGTTCGTGCCGGATCCCTTTG	pAF11
Overlap mCher/PvdI stop-700 Rev	CCTCCTCGCCCTTGCTCACGATCGCCTCTAGTTCGCTC	pAF11
Overlap mCher/PvdI stop+700 For	GCATGGACGAGCTGTACAAGTGACCCATGCTTTCCAATCCA	pAF11
PvdI-HindIIIRC	TTTAAGCTTGCCGGTCCAGTACGCCAACTG	pAF11
pvdJEcoRI-975F	TTTGAATTCGTGACGCCGCTGCTGTGG	pTS01
pvdJ/mcheOvR	CCTCCTCGCCCTTGCTCACGGAAATCAGTTTTTCAAGTTCATCGG	pTS01
pvdJHindIII+905R	AAAAAGCTTTGCCCGCTGTAGCGATACAG	pTS01
mChe/PvdJovF	GCATGGACGAGCTGTACAAGTAAGAGGCGGTAGCGTGCAAG	pTS01
FpvDEcoRI -943pb forw	TTTGAATTCCCTACATGCCGATCGGTACG	pTS04
PAmcherry/pvdD OvR	CCTCCTCGCCCTTGCTCACCACGCTACCGCCTCTTAGG	pTS04
pvdD/PA mcherry OvF	GCATGGACGAGCTGTACAAGCAAGCACTCATAGAGAAGGTGG	pTS04
pvdD-HindIII+934 pb rev	AAAAAGCTTGCACGCCGACACGGATATC	pTS04

Table S2: Oligonucleotides used to construct plasmids

Figure S1

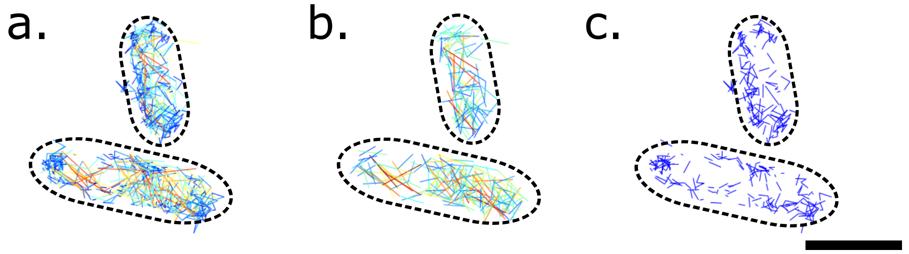

Figure S1: Representative diffusion maps of PvdD (the scale bar = 1 μm) made of **a.** all the reconstructed trajectories, **b.** trajectories filtered by a median jump distance > 0.2 μm or **c.** trajectories with median jump distances shorter than 0.15 μm.
